# Sarcopenia, Malnutrition, and Cachexia: Adapting Definitions and Terminology of Nutritional Disorders in Older People with Cancer

**DOI:** 10.3390/nu13030761

**Published:** 2021-02-26

**Authors:** Delky Meza-Valderrama, Ester Marco, Vanesa Dávalos-Yerovi, Maria Dolors Muns, Marta Tejero-Sánchez, Esther Duarte, Dolores Sánchez-Rodríguez

**Affiliations:** 1 Rehabilitation Research Group, Hospital del Mar Medical Research Institute (IMIM), Dr. Aiguader, 88, 08003 Barcelona, Catalonia, Spain; dmezaconcepcion@psmar.cat (D.M.-V.); emarco@parcdesalutmar.cat (E.M.); ndavalos@parcdesalutmar.cat (V.D.-Y.); mtejero@parcdesalutmar.cat (M.T.-S.); eduarte@parcdesalutmar.cat (E.D.); 2 PhD Program in Biomedicine, Department of Experimental and Health Sciences, Universitat Pompeu Fabra—Doctoral School, Dr. Aiguader, 88, 08003 Barcelona, Catalonia, Spain; 3 Physical Medicine and Rehabilitation Department, National Institute of Physical Medicine and Rehabilitation (INMFRE), Vía Centenario, Diagonal a la Universidad Tecnológica de Panamá, Panama City 0819, Panama; 4 Physical Medicine and Rehabilitation Department, Policlínica Manuel Váldes, Caja de Seguro Social (C.S.S.), Calle de Circunvalacion, Panama City 0844, Panama; 5 Physical Medicine and Rehabilitation Department, Hospital de la Esperança-Parc de Salut Mar, Sant Josep de la Muntanya 12, 08024 Barcelona, Catalonia, Spain; 6 School of Medicine, Universitat Autònoma de Barcelona, Bellaterra, 08193 Barcelona, Catalonia, Spain; 7 School of Medicine, Universitat International de Catalunya, c/Immaculada, 22, 08017 Barcelona, Catalonia, Spain; 8 Endocrinology and Nutrition Department, Hospital del Mar-Parc de Salut Mar, Passeig Marítim de la Barceloneta, 25, 29, 08003 Barcelona, Catalonia, Spain; mmuns@psmar.cat; 9 Clinical Research Unit, CHU Brugmann, Place Van Gehuchten 4, 1020 Brussels, Belgium; 10 Geriatrics Department, Centro Forum-Parc de Salut Mar, Llull 410, 08029 Barcelona, Catalonia, Spain; 11 WHO Collaborating Centre for Public Health Aspects of Musculo-Skeletal Health and Ageing, Division of Public Health, Epidemiology and Health Economics, University of Liège, CHU, Sart Tilman, Quartier Hôpital, Avenue Hippocrate 13 (Bât. B23), 4000 Liège, Belgium

**Keywords:** sarcopenia, malnutrition, cachexia, cancer, muscle mass, older people.

## Abstract

The recent publication of the revised Consensus on definition and diagnosis of sarcopenia (EWGSOP2) and the Global Leadership Initiative on Malnutrition (GLIM) criteria changed the approach to research on sarcopenia and malnutrition. Whilst sarcopenia is a nutrition-related disease, malnutrition and cachexia are nutritional disorders sharing the common feature of low fat-free mass. However, they have differential characteristics and etiologies, as well as specific therapeutic approaches. Applying the current definitions in clinical practice is still a challenge for health professionals and the potential for misdiagnosis is high. This is of special concern in the subgroup of older people with cancer, in which sarcopenia, malnutrition, and cancer cachexia are highly prevalent and can overlap or occur separately. The purpose of this review is to provide an updated overview of the latest research and consensus definitions of sarcopenia, malnutrition, and cachexia and to discuss their implications for clinical practice in older patients with cancer. The overall aim is to improve the quality of nutritional care in light of the latest findings.

## 1. Introduction

Sarcopenia, malnutrition, and cachexia may occur within a wide range of diseases, and their presence is associated with poorer health outcomes in all populations; all three are highly prevalent in older patients with cancer [1,2,3]. The potential for misdiagnosis is high because they share certain characteristics and overlap in some of their criteria; however, their physiopathology, etiology, and prognosis differ widely, as do diagnostic and therapeutic approaches. The harmonization of international terminologies, definitions, and diagnostic criteria of sarcopenia, malnutrition, and cachexia, as well as the early implementation of therapeutic approaches as part of the standard of care in clinical practice, will require a collaborative effort and must not be delayed. However, the overlapping criteria contained in the latest definitions makes it challenging to applying the definitions in clinical practice.

An initial question is whether sarcopenia, malnutrition, and cachexia are to be defined as diseases, disorders, syndromes, or conditions—terms often used interchangeably, but having different meanings. A disease is any deviation from or interruption of the normal structure or function of an organ or system of the body as manifested by characteristic symptoms and signs [4]. A disorder is defined as a derangement or abnormality of function; a morbid physical or mental state [4]. A syndrome is a complex of signs and symptoms resulting from a common cause or appearing, in combination, to present a clinical picture of a disease or inherited abnormality [5]. Finally, condition indicates a state of physical and mental health or well-being. The illness defined as a condition might be further classified as a disease or a disorder. However, the term condition also might be used in place of disease or disorder when a value-neutral term is desired [5,6].

Sarcopenia was initially considered a geriatric syndrome by EWGSOP in 2010 [7], as well as a nutrition-related condition by the European Society of Clinical Nutrition and Metabolism (ESPEN) [8]. The inclusion of a disease in the International Classification of Diseases (ICD-10) has implications in terms of clinical practice and healthcare costs [9,10]. Particularly in the case of sarcopenia, this inclusion has led to the EWGSOP2 consideration of sarcopenia as a muscle disease related to age (primary sarcopenia), but also to other diseases (secondary sarcopenia). The ESPEN guidelines consider malnutrition and undernutrition as synonyms and define them as nutritional disorders [8]. Cachexia has been defined as a multifactorial syndrome associated with underlying illness [11,12]; more recently, cachexia has been conceptualized as a type of disease-related malnutrition associated with chronic inflammation that should not be perceived as an end-stage of malnutrition [8]. 

Regardless of the term used (disease, disorder, illness, syndrome, or condition), there may be multiple pathogenic backgrounds and differences in impact on specific populations (e.g., older patients with cancer). Sarcopenia, malnutrition, and cachexia share the common feature of low fat-free mass, but they have differential characteristics, etiologies, and treatments. 

Other questions also arise: Should we approach age-related sarcopenia differently than sarcopenia related to diseases in older patients with cancer? Should we recommend physical activity to all patients with cancer, even those having a negative energy balance? A good understanding of these nutrition-related conditions, including definitions, screening tools, and diagnostic criteria, is the first step towards being able to answer these questions.

The purpose of this review is to provide an updated overview of the latest research and consensus definitions of sarcopenia, malnutrition, and cachexia, and to discuss their implications for clinical practice in older patients with cancer.

## 2. Sarcopenia

Sarcopenia in cancer patients has been associated with poorer quality of life, depression [13], and adverse clinical outcomes [14]. Furthermore, sarcopenia is highly correlated with the incidence of severe chemotherapeutic toxicity and associated changes in body composition, including the loss of skeletal muscle mass secondary to oncospecific treatments [15,16,17]. Survival in older patients with cancer can be affected by a combination of factors: Increased vulnerability to adverse outcomes secondary to cancer treatment, reduced physical reserve, and in some cases, the impossibility of further cancer-directed treatment [18]. Early assessment could benefit all older patients at risk of developing sarcopenia. Validated screening tools have emerged for this purpose, being the SARC-F (Strength, Assistance in walking, Rise from chair, Climb stairs, and Falls) the most commonly recommended questionnaire to identify people at risk of developing sarcopenia-associated adverse outcomes [19,20,21]. 

**Figure 1:**
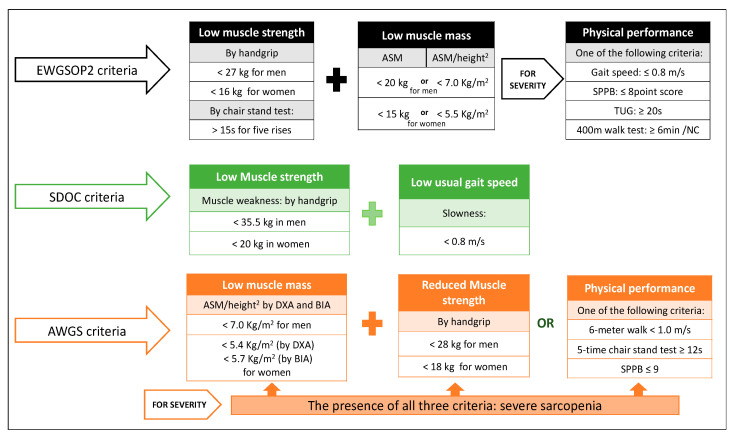
Update of the sarcopenia diagnostic criteria. Abbreviations: EWGSOP2: European Working Group on Sarcopenia in Older People 2; AWGS: Asian Working Group for Sarcopenia; SDOC: Sarcopenia Definition and Outcomes Consortium; ASM: Appendicular Skeletal Muscle Mass; SPPB: Short Physical Performance Battery; TUG: Timed-Up and Go test; NC: Non-completion. DXA: Dual-energy X-ray Absorptiometry. BIA: Bioelectrical Impedance Analysis [20,23,25].

Since the term sarcopenia began to be used more than 20 years ago, researchers have tried to reach agreement on its definition: Is it an age-related loss of muscle mass and function, a disease, or a process of normal aging? [22]. The European Working Group on Sarcopenia in Older People 2 (EWGSOP2) defines sarcopenia as a muscle disease (muscle failure) rooted in adverse muscle changes that accrue across a lifetime, associated with a higher probability of adverse outcomes such as falls, fractures, physical disability, and mortality [20]. Other international initiatives led by the Asian Working Group for Sarcopenia (AWGS) and the Sarcopenia Definition and Outcomes Consortium (SDOC) have launched new diagnostic criteria based on the scientific evidence obtained over the years [20,23,24], but a universal consensus is still lacking. Since muscle strength has a greater capacity than muscle mass to predict poor outcome in patients with sarcopenia, the EWGSOP2 guidelines point out the loss of muscle strength as the most relevant criterion for its diagnosis. Hence, sarcopenia must be suspected in the presence of low muscle strength and confirmed by documentation of loss of skeletal muscle mass (Figure 1). The EWGSOP2 distinguishes between primary sarcopenia (age-related) and secondary sarcopenia (in the presence of underlying systemic disease or inflammatory processes) and provides specific cut-off points for (1) low muscle strength and (2) low muscle mass. Gait speed, the Short Physical Performance Battery (SPPB), or the Timed Get-Up and Go test, commonly used to assess physical performance, become indicators of the severity of the disease once it is diagnosed [20,23,25].

The Foundation for the National Institutes of Health (FNIH) Biomarkers Consortium published a series of manuscripts framed under “The FNIH Sarcopenia Project” with specific recommendations on cut-off points for weakness and low appendicular lean muscle mass [26]; the EWGSOP2 later used very similar cut-off points [20,26,27]. In 2016, to address the need for a refined and updated operational definition of sarcopenia, the FNIH and the National Institute of Aging funded the SDOC. This consortium, in its 2020 position statements, considers that muscle weakness, defined by low grip strength (<35.5 kg in men or 20 kg in women), and slowness (usual gait speed <0.8 m/s) are sufficient criteria to diagnose sarcopenia [24,25]. The most controversial SDOC recommendation is to exclude lean mass measured by dual-energy x-ray absorptiometry (DXA) from the sarcopenia definition [25]. The SDOC states that both low grip strength and low usual gait speed are independent predictive factors of falls, self-reported mobility limitation, hip fractures, and mortality in community-dwelling older adults; therefore, they should be included in the definition of sarcopenia. However, lean mass measured by DXA was not associated with incident adverse health-related outcomes.

Finally, the AWGS, in its 2019 consensus update, maintains the original definition of sarcopenia as an age-related loss of skeletal muscle mass accompanied by low muscle strength and/or physical performance [23]. The presence of all three criteria corresponds to severe sarcopenia (Figure 1).

Advances in the diagnosis and treatment of sarcopenia and the evidence supporting its association with adverse outcomes in older people establish that sarcopenia is an age-related muscle disease [9,20,23,24] rather than simply a normal physiologic process of aging [28]. It is important to emphasize that approaching sarcopenia as a normal part of aging may result in a misinterpretation of the current definition. The adequate qualitative and quantitative measurement of muscle mass remains a challenge, particularly for older patients with cancer, and no consensus has been reached on the use and interpretation of these measurements [29]. 

## 3. Malnutrition

Malnutrition has been described as a state resulting from lack of intake or uptake of nutrients that leads to altered body composition and body cell mass, resulting in impaired physical and mental function [8,30,31]. The ESPEN guidelines on definitions and terminology of clinical nutrition provides an etiology-based approach, distinguishing among disease-related malnutrition (DRM) with inflammation, DRM without inflammation, and malnutrition without disease [8,32]. Societies such as the American Society for Parenteral and Enteral Nutrition (ASPEN) and the ESPEN developed clinical guidelines that recommend the use of screening tools for early detection and treatment of nutritional disorders [33,34]; the Mini Nutritional Assessment Short Form (MNA-SF) is considered one of the major malnutrition screening tools in older adults [35,36]. 

One of the main features of malnutrition is involuntary weight loss, which is associated with an increased likelihood of post-discharge institutionalization [37]. Regardless of the importance of weight loss, malnutrition should be addressed as a muscle-related disorder, including a proper skeletal muscle assessment in clinical practice [38]. The Academy of Nutrition and Dietetics (AND) and ASPEN emphasize that no single parameter is definitive for adult malnutrition diagnosis and recommend assessing energy intake, weight loss, muscle mass, subcutaneous fat, fluid accumulation, and muscle strength [32]. Subsequently, the ESPEN launched two diagnostic criteria based on three variables: Weight loss, reduced body mass index (BMI), and reduced fat-free mass index [31]. Even though diagnostic criteria were associated with a longer length of hospital stay, the prevalence of malnutrition in post-acute care was very different when compared using the AND/ASPEN and ESPEN proposals [39]. 

**Figure 2:**
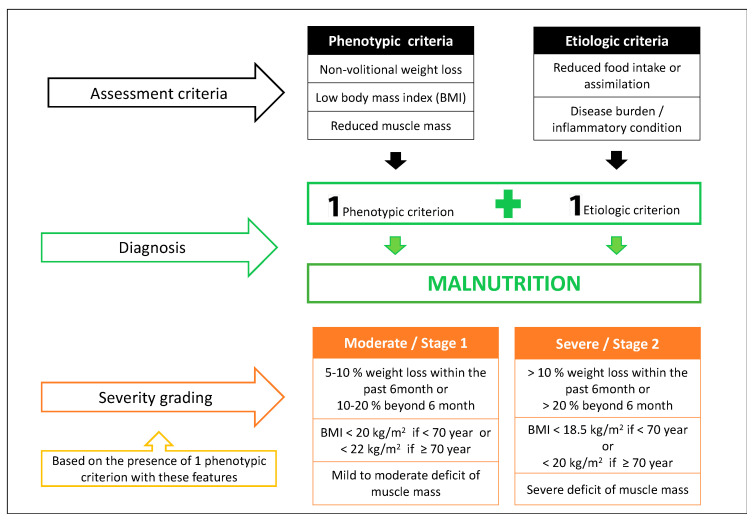
The Global Leadership Initiative on Malnutrition (GLIM) criteria of malnutrition diagnosis and severity grading [36].

Given the lack of a worldwide consensus on diagnostic criteria, together with new evidence supporting the influence of disease and inflammation on malnutrition, the Global Leadership Initiative on Malnutrition (GLIM) engaged the majority of nutrition societies in an effort to standardize the diagnosis of malnutrition in clinical settings [36,40]. The GLIM proposes a three-step approach: First, patients must be identified by a validated screening tool; second, malnutrition requires the presence of at least one phenotypical criterion and one etiological criterion; and finally, severity is based on threshold levels of the phenotypic criteria (Figure 2).

In patients with cancer, the assessment of malnutrition should be a fundamental and mandatory part of the clinical evaluation, since nutritional and metabolic disorders are associated with a negative effect on clinical outcomes [1,41] such as a longer length of hospital stay [42], increased infection and hospital readmissions [43], postoperative complications [42,44], and mortality [43,45,46,47]. The ESPEN guidelines on nutrition in cancer patients launched in 2016 aimed to provide precise recommendations for the multimodal nutritional management [41]. Shortly thereafter, the ESPEN expert group launched recommendations for action against cancer-related malnutrition to facilitate nutrition support in clinical practice for the care of patients with cancer. Three key points were given: (1) Screen all patients with cancer, (2) expand nutrition-related assessment practices, and (3) use multimodal nutritional interventions with individualized plans [1]. All these initiatives seek to raise awareness among health professionals about the importance of assessing malnutrition in patients with cancer, as well as providing timely measures of nutritional support that help improve outcomes in cancer patients.

## 4. Cancer Cachexia

**Figure 3:**
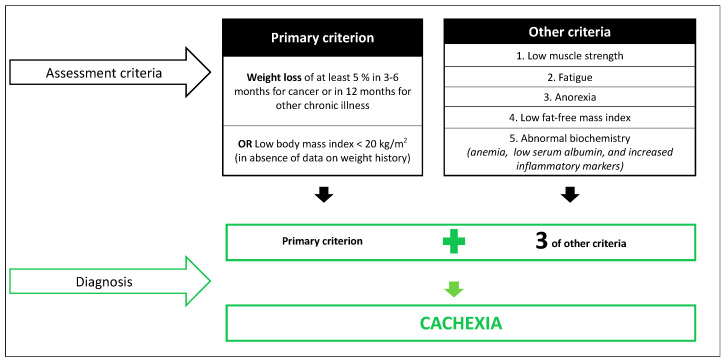
Cachexia diagnostic criteria by Evans et al. [11].

Cachexia is a common manifestation of several serious illnesses, such as chronic heart failure, acquired immune deficiency syndrome, and cancer [48]. Cachexia and, more specifically, cancer cachexia is a type of disease-related malnutrition associated with chronic inflammation, which should not be perceived as end-stage malnutrition [8]. Reaching a single definition with specific diagnostic criteria is still a challenge for the scientific community [11,12,49,50]. One of the first successful attempts was achieved in the Cachexia Consensus Conference held in Washington DC in December 2006 [11]. Cachexia was defined as “a complex metabolic syndrome associated with underlying illness and characterized by loss of muscle with or without loss of fat mass”, where weight loss was pointed out as the most important feature of cachexia in adults. Diagnostic criteria included weight loss as a primary criterion, plus three of five other criteria (Figure 3). In the absence of data on weight history, this consensus recommends the use of BMI < 20 kg/m^2^ as primary criterion [11]. These criteria required specific equipment to assess muscle strength and body composition, as well as blood testing, which might have limited the use of this definition in clinical practice.

The SCRINIO Working Group proposed simpler criteria based on the loss of at least 10% of body weight and the presence of one of the associated symptoms (fatigue, anorexia, or early satiation) [51]. These criteria allow ready identification of patients with cancer cachexia and classification as precachexia or cachexia [51]; this practical approach has improved applicability [52].

**Figure 4:**
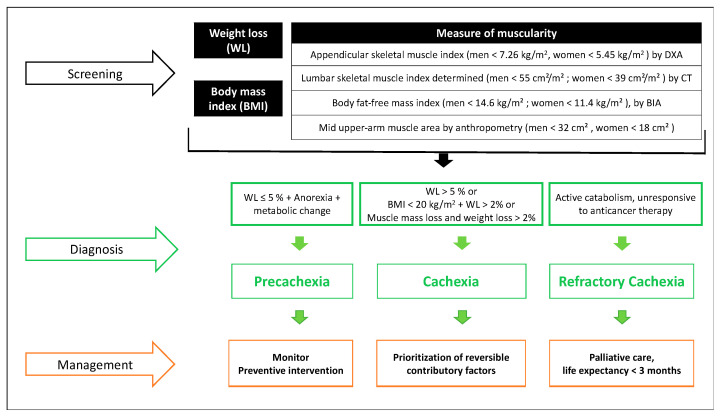
Cancer cachexia approach by Fearon et al. [12]. Abbreviations: DXA: Dual-energy X-ray Absorptiometry. CT: Computed Tomography. BIA: Bioelectrical Impedance Analysis.

In 2012, an international consensus process was initiated to reach a more specific definition of cancer cachexia, involving an expert panel of the European Palliative Care Research Collaborative, the Society on Cachexia and Wasting Disorders, the National Cancer Research Institute Palliative Care Clinical Studies Group, and the European Society for Clinical Nutrition and Metabolism Special Interest Group on Cachexia. Cancer cachexia was defined as a multifactorial syndrome characterized by an ongoing loss of skeletal muscle mass (with or without loss of fat mass) that cannot be fully reversed by conventional nutritional support and leads to progressive functional impairment [12]. One of the critical aspects introduced by this consensus was the concept of progressivity of cancer cachexia, which prioritizes the early search for signs that may indicate a negative protein and energy balance. The classification into three stages became especially important due to their respective therapeutic implications (Figure 4): Pre-cachexia represents the greatest opportunity for preventive interventions; cachexia involves multimodal management, focused on reversible contributory factors; and refractory cachexia is understood as the stage where there is no response to anticancer treatments, with active catabolism and a life expectancy of fewer than three months. Refractory cachexia is part of palliative care aimed to relieve symptoms and provide the necessary support for patients and families [12]. Not all patients go through all three stages: Progression depends on factors such as the severity of the oncological process, the level of systemic inflammation, reduced food intake, and lack of response to anticancer therapy [12].

All these criteria should be appropriate to detect cancer cachexia in older people, as they can identify patients with a higher mortality risk [2]. However, their pros and cons deserve some consideration. While the criteria of Evans have shown a stronger predictive capacity on the overall survival of patients with cancer [53], other diagnostic criteria such as those of Fearon and the SCRINIO Working Group seem to be more feasible for the systematic bedside assessment of cachexia in daily clinical practice [2]. The common parameters in the different and most accepted criteria proposed today are weight loss and anorexia [2,11,12,51,52,53]. Therefore, screening for these parameters should be a fundamental part of the initial assessment and follow-up of all older patients with and without cancer.

## 5. Overlap and Differential Diagnosis: Upcoming Landmarks and Points for Further Discussion

Sarcopenia and cachexia can occur concurrently in the same patient [54]; this is associated with poor outcomes, especially in older patients with cancer [2,55]. The etiology of muscle-wasting that seems to be present in both sarcopenia and cachexia has distinct mechanisms [56]; however, differential diagnosis might be difficult in clinical practice, as there is no clear demarcation line between these two entities [57] or screening tools to distinguish between them [58,59]. Therefore, overlap is possible [28,57]. 

The loss of muscle mass is the common feature shared by sarcopenia and cachexia. Whilst the EWGSOP and the AWGS require both low muscle mass and decreased strength for the diagnosis of sarcopenia, Evans et al. propose the presence of decreased muscle strength and/or low fat-free mass for the cachexia diagnosis [11], and Fearon et al. suggest sarcopenia as a diagnostic criterion for cancer cachexia [12]. While involuntary weight loss and reduced food intake (anorexia) in the context of chronic systemic inflammation and metabolic alterations are important features of cachexia [11,12,49], sarcopenia is an age-related disease where weight loss is not a diagnostic criterion [20]. 

The continuous search for clear concepts that lead to a correct evaluation of nutritional status in older people is increasingly important in clinical practice. Nutrition-related conditions such as sarcopenia and nutrition disorders such as malnutrition and cachexia are common in patients with cancer and even more so in the oldest patients [1,2,3]. However, while malnutrition underlies an imbalance between energy intake, energy expenditure, and the quality of the nutrient intake [8,36] and, as in cachexia, it may be associated with a disease with inflammatory activity [36], sarcopenia is a progressive and generalized skeletal muscle disorder, which may or may not be associated with another disease or an inflammatory process [20]. Nonetheless, decreased muscle mass is one of the foremost features of sarcopenia, as in malnutrition and cachexia. In addition, malnutrition is a strong predictor of sarcopenia and severe sarcopenia [60]. Therefore, achieving adequate prevention and treatment measures is only possible if all these clinical concepts are sufficiently clear at the time of assessment, especially if an oncological process is involved.

This review provides information on similarities, differences, and possible overlap of sarcopenia, malnutrition, and cancer cachexia in older patients with cancer. Changes in body composition and their impact on nutritional status require further study.

## 6. Summary

Adequate management of the patient with cancer requires assessment of muscle function and nutritional status. In older patients with cancer, the use of validated screening tools, the follow-up of patients at risk, and accurate early diagnosis of malnutrition, cachexia, and/or sarcopenia are the pillars for timely treatment to improve clinical outcomes. There is still a lack of agreement, mainly in the criteria and diagnostic cut-off points for these three entities. However, current research has provided tools to help health professionals make an early diagnosis and prescribe treatment. Just as the research community is called to achieve consensus on definitions and diagnostic criteria applicable in clinical practice, physicians and other health professionals are called to put into practice the updated guidelines on sarcopenia, malnutrition, and cachexia.
